# A Wide Energy Range and 4π-View Gamma Camera with Interspaced Position-Sensitive Scintillator Array and Embedded Heavy Metal Bars

**DOI:** 10.3390/s23020953

**Published:** 2023-01-13

**Authors:** Yifan Hu, Zhenlei Lyu, Peng Fan, Tianpeng Xu, Shi Wang, Yaqiang Liu, Tianyu Ma

**Affiliations:** 1 Department of Engineering Physics, Tsinghua University, Beijing 100084, China; 2 Key Laboratory of Particle & Radiation Imaging, Ministry of Education, Beijing 100084, China; 3 Institute for Precision Medicine, Tsinghua University, Beijing 100084, China; 4 Beijing Institute of Spacecraft System Engineering, Beijing 100094, China; 5 State Nuclear Security Technology Center, Beijing 102401, China

**Keywords:** industrial gamma camera, high resolution, scintillation detector, collimation

## Abstract

(1) Background: Gamma cameras have wide applications in industry, including nuclear power plant monitoring, emergency response, and homeland security. The desirable properties of a gamma camera include small weight, good resolution, large field of view (FOV), and wide imageable source energy range. Compton cameras can have a 4π FOV but have limited sensitivity at low energy. Coded-aperture gamma cameras are operatable at a wide photon energy range but typically have a limited FOV and increased weight due to the thick heavy metal collimators and shielding. In our lab, we previously proposed a 4π-view gamma imaging approach with a 3D position-sensitive detector, with which each detector element acts as the collimator for other detector elements. We presented promising imaging performance for ^99m^Tc, ^18^F, and ^137^Cs sources. However, the imaging performance for middle- and high-energy sources requires further improvement. (2) Methods: In this study, we present a new gamma camera design to achieve satisfactory imaging performance in a wide gamma energy range. The proposed gamma camera consists of interspaced bar-shaped GAGG (Ce) crystals and tungsten absorbers. The metal bars enhance collimation for high-energy gamma photons without sacrificing the FOV. We assembled a gamma camera prototype and conducted experiments to evaluate the gamma camera’s performance for imaging ^57^Co, ^137^Cs, and ^60^Co point sources. (3) Results: Results show that the proposed gamma camera achieves a positioning accuracy of <3° for all gamma energies. It can clearly resolve two ^137^Cs point sources with 10° separation, two ^57^Co and two ^60^Co point sources with 20° separation, as well as a 2 × 3 ^137^Cs point-source array with 20° separation. (4) Conclusions: We conclude that the proposed gamma camera design has comprehensive merits, including portability, 4π-view FOV, and good angular resolution across a wide energy range. The presented approach has promising potential in nuclear security applications.

## 1. Introduction

The industrial gamma cameras enable visualization of the gamma source distribution at a standoff distance (e.g., several meters to tens of meters), acceleration of the gamma material localization process, and reduction in radiation exposure risk [1]. To accurately and efficiently accomplish the imaging task, the following system performance requirements are desired for an industrial gamma camera: (1) a large field of view (FOV), which enables quick locating of radiation risk and reduces the searching time and radiation exposure; (2) a wide energy range, covering the frequently used industrial gamma isotopes including ^241^Am (59.5 keV), ^57^Co (122 keV and 136 keV), ^99m^Tc (140 keV), ^133^Ba (356 keV), ^22^Na (511 keV and 1275 keV), ^137^Cs (662 keV), and ^60^Co (1173 keV and 1333 keV); (3) a high positioning accuracy to rapidly locate the radiation hazard, improve operational efficiency, and minimize the risk of improper operation due to mispositioning of the radiation hotspot, e.g., in homeland security applications; (4) and a compact size that facilitates use in various application scenarios.

Conventional industrial gamma cameras include coded-aperture gamma cameras [2] and Compton cameras [3]. Coded-aperture gamma cameras use a mechanical collimator to image gamma rays in a limited FOV [4]. In principle, this technology is operational over a wide energy range. However, thicker metal collimators and shielding are needed for higher energy gamma rays, which significantly increase the weight [5]. Compton cameras can achieve up to 4π-view FOV with a high detection sensitivity [6]. However, they are usually not capable of imaging low-energy sources (<200 keV) due to the low interaction probability [7]. Several recent works also equip Compton cameras with a metal coded-aperture collimator [8] to image low-energy isotopes in a reduced FOV, or with an active pinhole detector layer [9], or a coded-aperture shape detector layer [10] to both expand the image FOV and energy range, but at the expense of increased system design complexity, device weight, and cost.

Our lab recently proposed a 4π-view gamma imaging approach using a collimatorless, 3D position-sensitive detector [11]. We propose a novel idea that each detector element plays a role of collimation for other detector elements. Hence, the measured photon distribution is sensitive to incoming gamma rays’ direction in 4π space. Unlike the Compton camera, the proposed approach only requires the knowledge of a single interaction position that accommodates different types of photons interaction and, in principle, allows 4π-view gamma imaging in a wide photon energy range. Another advantage is that the proposed method does not critically rely on high detector energy resolution as the Compton camera does. We demonstrated a significant resolution improvement in [12] with an advanced interspaced, mosaic-patterned detector element matrix design. However, one challenge in this approach is that when each detector element plays the collimation role for others, the efficacy of collimation also depends on the incoming photon’s energy. For high-energy photons, scintillators have lower stopping power, which increases photon penetration possibility, weakens the collimation effect, and further degrades imaging performance. A larger detector size is desired to enhance the detector–collimation effect, leading to increased system cost and less compact size.

In this work, we propose a new gamma camera design that hybrids the mechanical collimation and our proposed detector collimation, aiming to enhance the imaging performance over a wide energy range and maintain the merit of 4π-view FOV and compact size. Unlike conventional mechanical gamma cameras, we propose embedding heavy metal elements into the 3D position-sensitive detector. The metal bars enhance collimation for high-energy gamma photons without sacrificing the 4π FOV. We assemble a gamma camera prototype and present experimental results to evaluate the efficacy of the new gamma camera.

## 2. Design Concept

**Figure 1:**
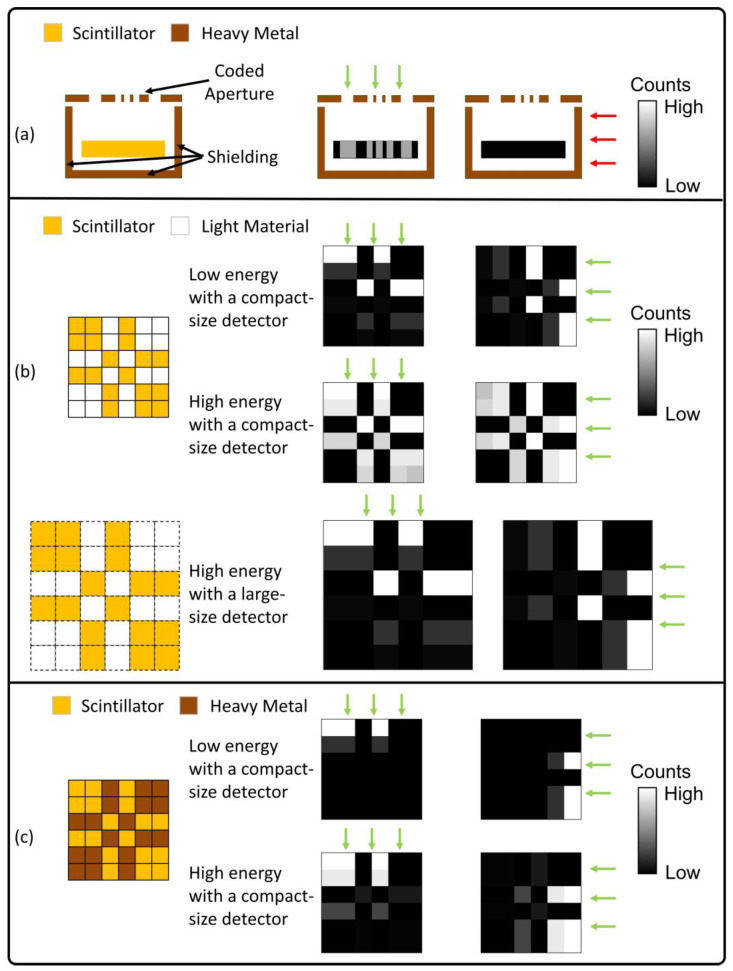
Gamma camera design concepts: (**a**) conventional gamma camera with a heavy metal collimator (**b**) previously proposed gamma camera using mosaic patterned scintillators and (**c**) proposed gamma camera using scintillators and embedded heavy metal elements.

The key concept of our proposed design is shown in Figure 1. Conventional coded-aperture gamma camera (Figure 1a) places a metal collimator between the incoming gamma rays and the scintillator detector. The projection data on the scintillator is determined by the gamma rays’ direction and can be used for gamma imaging. However, the target FOV is limited by the size of the collimator and detector, and the metal shielding on the side and back face increases weight.

Figure 1b shows our previously proposed collimatorless, 4π-view gamma camera design with mosaic-patterned scintillators [12]. The scintillators not only detect photons themselves but also induces collimation for other scintillators, producing photon event distributions sensitive to incoming gamma ray direction.

The uppermost and middle rows in Figure 1b compare the imaging ability of photons at different energy. For a compact-sized detector, when the photon energy increases, the increased penetration through scintillators causes less dependence of photon event distribution on the gamma ray direction, implying degraded imaging performance. As shown in the lowermost row in Figure 1b, it is possible to enhance the scintillator-induced collimator by using a larger scintillator size, but at the cost of increased detector size.

The proposed design in this work is shown in Figure 1c. We embedded separate heavy metal elements into the interspaced scintillator array. For low-energy gamma sources, the gamma rays deposit most of their energies in outer scintillators, inducing a similar photon-collimation effect as the scintillator-only design in Figure 1b. For high-energy gamma sources, the embedded heavy metal elements absorb a portion of gamma photon (which could penetrate the detector in the previous scintillator-only design) and enhance direction dependency of photon events distribution. This design can improve high-energy imaging performance for photons and retain the merit of 4π-view FOV and compact size.

## 3. Gamma Camera Development and Experiments

### 3.1. The Gamma Camera

**Figure 2:**
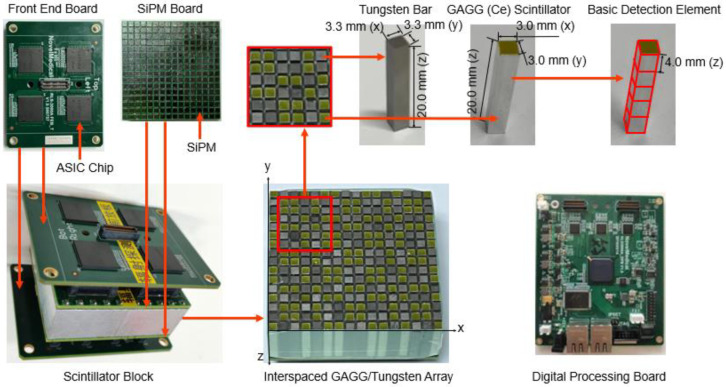
Gamma camera components: detector module and readout electronics.

As shown in Figure 2, the detection module of the gamma camera is composed of 128 interspaced GAGG (Ce) crystals and 128 tungsten bars. Each GAGG (Ce) crystal has a size of 3.0 mm (x) × 3.0 mm (y) × 20.0 mm (z) and is wrapped with a 0.15 mm thickness BaSO_4_ reflector (Epic Crystal, China). Each tungsten bar has a size of 3.3 mm (x) × 3.3 mm (y) × 20.0 mm (z). We chose the size to make the bar-shaped GAGG (Ce) with reflector and tungsten have the same cross sections. The array is optically coupled to two SiPM (Onsemi, FJ30035) boards at both ends. 

**Figure 3:**
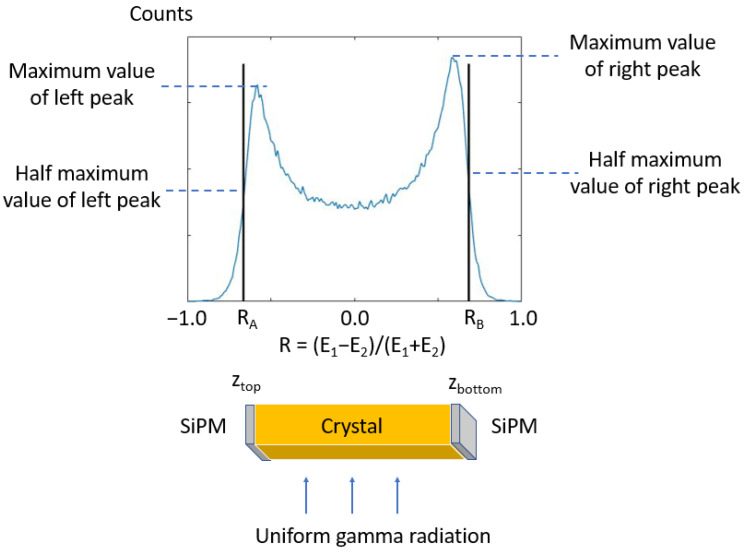
Illustration of *z* position calibration method.

When an incoming photon deposits energy on a crystal bar, output signals of each SiPM array are fed to a self-developed ASIC chip [13] on the front-end board. The ASIC chip calculates 2D position signals X, Y using anger logic and energy signal E using waveform interpolation for each gamma event. The X, Y and E signals are then transferred to the digital processing board shown in Figure 2. The waveform of signals is digitized by an A/D converter (AD9637, 12 bit, and 80 MHz sampling rates). An FPGA (Spantan-6, Xilinx) performs signal and data control. Finally, digitized signals are sent to a computer using a cable. We use X and Y signals to determine the interaction crystal bar. Then we used the energy signal of two SiPM arrays to calculate the interaction *z* position in each crystal bar [14,15]:

$$z=k\frac{E_{1}-E_{2}}{E_{1}+E_{2}}+b,$$

where *E*_1_ and *E*_2_ are the energy signals at both ends. The *z* position calibration is conducted with an uncollimated source that uniformly irradiates the crystal. Readers are referred to [14] for a detailed description of the calibration methodology. As a brief description, the calibration steps are:

(1) Measure the SiPM signals *E*_1_ and *E*_2_ on both ends and assume a linear relationship between $R=\frac{E_{1}-E_{2}}{E_{1}+E_{2}}$ and the *z* position, i.e., z=kR+b;
(2) Irradiate the crystal with an uncollimated flood source and measure a histogram of R as shown in Figure 3;
(3) Find two points *A* and *B*, which are on the histogram curve’s left and right falling edges and with *R_A_* and *R_B_* values being 1/2 of the maximum values of the left and right peaks;
(4) By assuming that these two points correspond to the gamma interactions on the top and bottom side of the crystal, calculate parameters *k* and *b* from the following equations:

*Z*_top_**=***k R*_*A*_ + *b*, and *Z*_bottom_ = *k R*_*B*_ + *b*.

With this method, the impact of mismatched SiPM gain can be incorporated in the calibration process for parameter *b*.

We virtually divided each crystal bar into five basic detection elements with a size of 3.0 mm (x) × 3.0 mm (y) × 4.0 mm (z). With the measured X, Y, and Z positions, we assemble each gamma event into corresponding basic detection elements. The bin size in z direction is chosen according to our previous studies [12].

### 3.2. Imaging Reconstruction

The imaging problem can be modeled using the below equation:

y=Px,

where ***x*** and ***y*** are column vectors and ***P*** is a 2D matrix. ***x*** represents the gamma radiation intensity in the 4π FOV, ***y*** is the projection data, ***P*** is the system matrix with each element {*p_ij_*} indicating the probability that a photon emitted from *jth* image pixel is detected in the *ith* detector bin.

We used a maximum-likelihood expectation-maximization (MLEM) algorithm for iterative update [16]:

$$x^{k+1}=\frac{x^{k}}{P^{T}1}\cdot \left(P^{T}\frac{y}{Px^{k}}\right),$$

where ***x****^k+1^* and ***x****^k^* represent reconstructed results at iteration *k* and *k* + 1, **1** is a column vector with all elements being 1.

**Figure 4:**
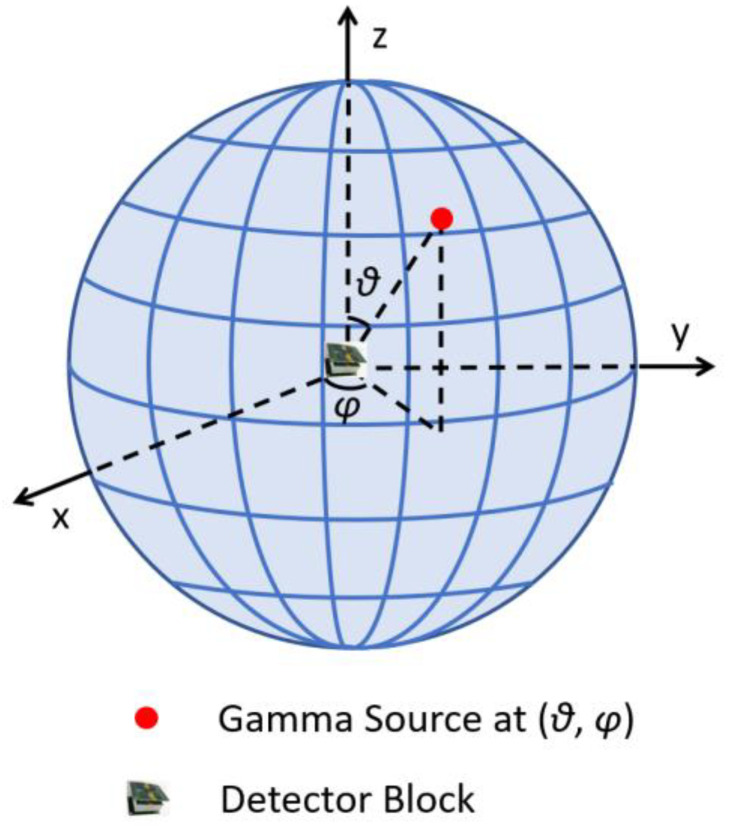
Definition of the spherical coordinate system in the 4π FOV.

As shown in Figure 4, we discretize the 4π space with a spherical angular coordinate system. The entire image has 181 (*θ*) × 360 (*φ*) pixels, with *θ* ranging from 0° to 180° and *φ* ranging from 0° to 359°. The pixel size is 1° × 1°. The projection data are defined as measured gamma photon counts in each detector element. As described in Section 3.1, there are 128 × 5 = 640 detection elements; therefore, ***y*** is a column vector with 640 elements.

### 3.3. Experiments

#### 3.3.1. Experimental Platform

**Figure 5:**
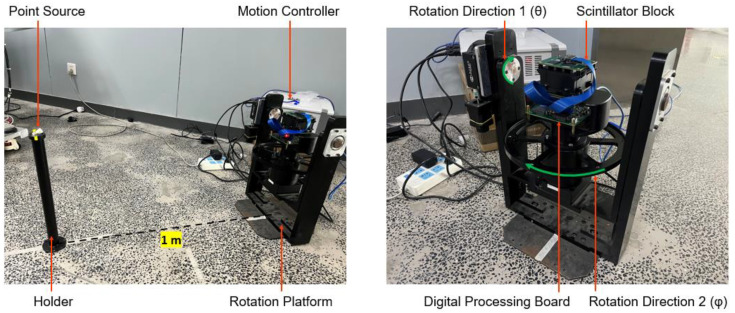
The experimental platform with two rotation axes that accurately control the relative direction between the detector block and the incoming gamma ray from the point source.

We assembled an experimental platform for imaging experiments. The platform can accurately control the relative direction (*θ*, *φ*) between the scintillator block and incoming ray from a fixed-point source. As shown in Figure 5, we mount the detector block on a rotational stage with two rotation axes. The distance between the gamma point source and the center of the detector block is ~1 m to warrant the far-field imaging geometry.

#### 3.3.2. Detector Calibration

We used ^57^Co, ^137^Cs, ^22^Na, and ^60^Co point sources to irradiate the top and bottom sides of the detector block. The ^57^Co, ^137^Cs, and ^60^Co sources are also used in imaging studies in Section 3.3.3, and the ^22^Na source with ~200 uCi activity is only used in detector calibration. We used the gamma events’ position signals (X, Y) to generate a 2D flood histogram. A self-developed software segments the flood histogram and creates the crystal look-up table [17]. The *z* position calibration is conducted using the uniform irradiation method described in Section 3.1. 

We measured the detector energy spectra for the four types of sources. We generated an energy spectrum for each individual crystal and found the energy peak position of each energy spectrum curve. We used the 122 keV peak of ^57^Co, 662 keV peak of ^137^Cs, and 511 keV and 1275 keV peak of ^22^Na to normalize all the spectrum curves of each source. In this way, the energy peak positions are aligned to a fixed position. Then we sum the normalized energy spectrums and generate the energy spectrums for the entire detector. To calculate the energy resolution, we select a range of +/− 20% around the 662 keV peak of ^137^Cs and 511 keV peak of ^22^Na, and +/− 10% around the 1275 keV peak of ^22^Na. We fit the curves within the range with gaussian functions. We reported the full width at half maximum (FWHM) energy resolution. Note that for the ^60^Co source, we applied the normalization factors derived from the 1275 keV peak of ^22^Na to avoid the down-scatter impact between the 1173 keV and 1333 keV peaks.

#### 3.3.3. System Matrix Measurement and Generation

We also used ^57^Co, ^137^Cs, and ^60^Co point sources to measure the system matrix in the 4π FOV. The activities of the radiation sources were measured in a qualified site. Due to limited measurement time and environmental conditions, we only measured a part of 4π FOV: *θ* varies from 0° to 90°, and *φ* varies from 0° to 180°. To further reduce the experimental time, we used a coarse grid: *θ* and *φ* have a 5° interval for ^57^Co and ^137^Cs experiments and a 10° interval for ^60^Co. The measurement parameters are listed in Table 1. The system matrices were generated using the method described in [18].

For ^57^Co and ^137^Cs, we expanded the measured system matrices from a 19 (θ) × 37 (φ) image grid (with a grid size of 5° × 5°) to a 181 (θ) × 360 (φ) image grid (with a grid size of 1° × 1°). We first interpolated the acquired system matrix in the image domain to that on a 91 (θ) × 180 (φ) image grid through cubic spline interpolation. Then we used geometrical symmetry to generate a full system matrix on a 181 (θ) × 360 (φ) image grid. For ^60^Co, the system matrix generation method is almost the same; the only difference is an interpolation from a 10° × 10° grid to a 1° × 1° grid.

#### 3.3.4. Point Source Imaging Experiments

We acquired the projection data at a series of positions with different counts in the FOV to evaluate the imaging performance. Details of measurement positions are listed in Section 4. We also combined the projection data at different positions to mimic the multiple source imaging cases.

## 4. Results

### 4.1. Detector Performance

**Figure 6:**
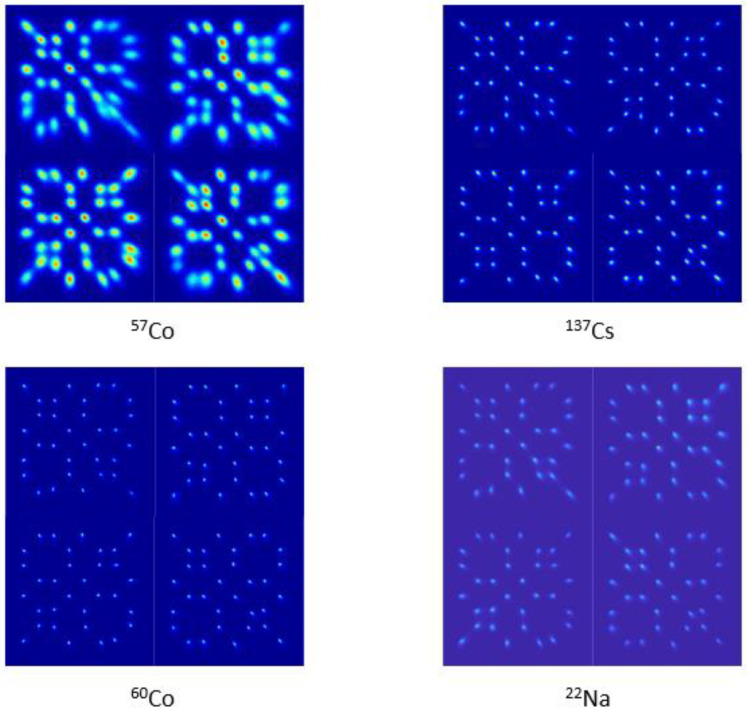
The detector’s measured flood histogram of ^57^Co, ^137^Cs, ^60^Co, and ^22^Na point sources.

Figure 6 shows the measured flood histogram of the detector block irradiated by ^57^Co, ^137^Cs, ^60^Co, and ^22^Na point sources. All the crystals are clearly resolved. Better crystal separation is observed with higher gamma ray energy because of the increased number of optical photons produced and less relative statistical fluctuation of the measured energy signal. 

**Figure 7:**
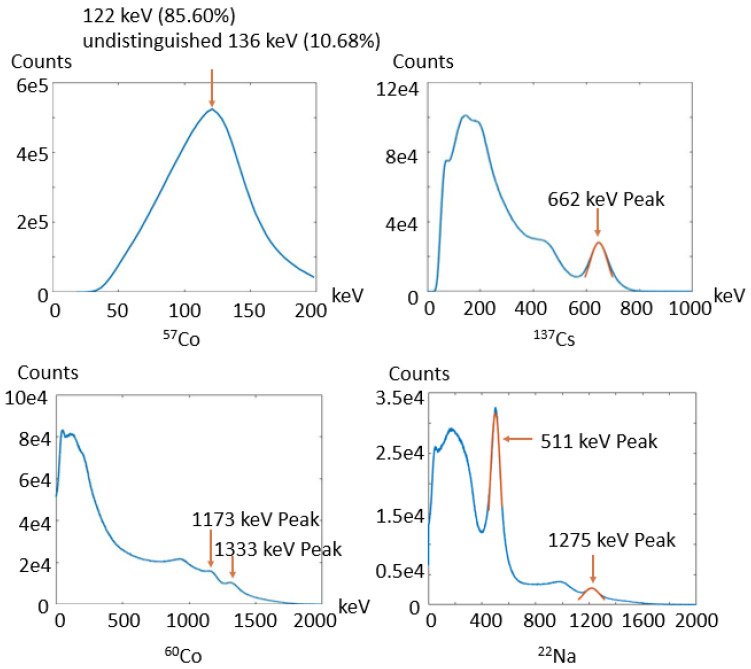
Energy spectrum in detector calibration (the blue line is the measured data and the red line is the Gaussian fitting of the energy peak).

Figure 7 shows the energy spectrum of the entire detector block. We normalized the energy spectrum of each crystal by aligning their energy peaks to a fixed value before calculating the average of all the energy spectrums. The measured energy resolution is 19.6% at 511 keV, 13.7% at 662 keV, and 11.8% at 1275 keV. The energy resolution at 662 keV is compared with other gamma cameras and spectrometers with GAGG (Ce) (5.6% in [19], 6.5% in [20], and 10.5% in [21]) and other commonly used scintillators such as NaI (Tl) (6.5% in [22] and 11% in [23]). We believe the dual-end-readout technique that spreads the optical photons over two SiPMs, the detector’s large crystal aspect ratio [24] and the diffuse reflector [15] contribute to the energy resolution degradation. The reported energy resolution at 511 keV is comparable to several published works with dual-end-readout GAGG (Ce) detectors (17.2% in [25] and 21.2% in [26]). Future work directions to refine the energy resolution are discussed in Section 5.

In the measured spectrum for ^57^Co, the energy peak is asymmetric. We believe it is due to the hybrid emission of 136 keV (10.68%) and 122 keV (85.6%) photons of ^57^Co. In the ^60^Co spectrum, the shape of energy peaks of 1173 keV and 1333 keV are impacted by each other due to relatively low energy resolution and down-scattered photons. For the above reasons, we did not perform energy spectrum fitting and energy resolution measurement for ^57^Co and ^60^Co sources.

**Figure 8:**
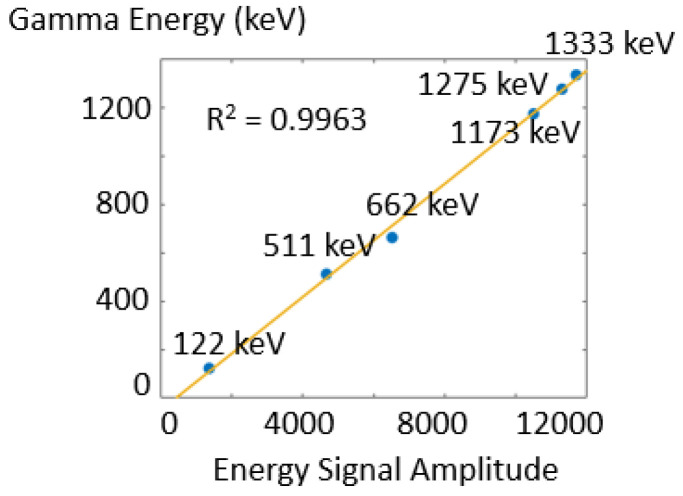
Energy response linearity in the single crystal and typical energy response fitting result.

In Figure 8 we evaluate the energy response linearity by showing the measured energy signal amplitude as a function of the corresponding gamma energy for one representative crystal. The energy response shows good linearity with R^2^ = 0.9963.

### 4.2. Single Point Source Imaging

**Figure 9:**
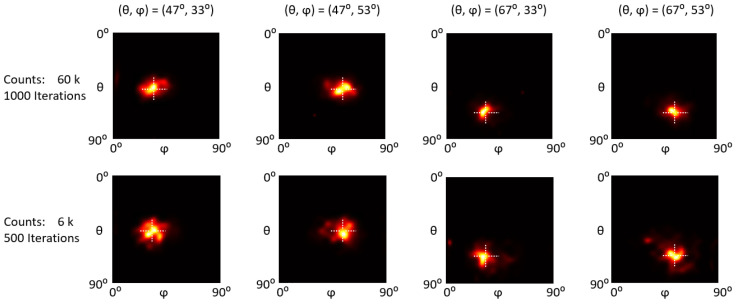
Reconstruction images of a ^57^Co point sources at different positions with different count levels. The intersections of the dotted lines indicate the actual positions.

**Figure 10:**
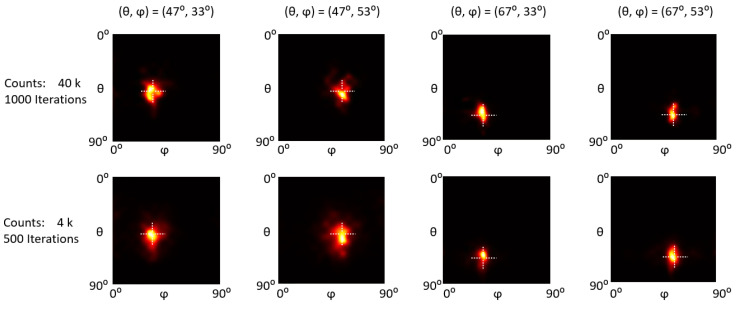
Reconstruction images of a ^137^Cs point sources at different positions with different count levels. The intersections of the dotted lines indicate the actual positions.

**Figure 11:**
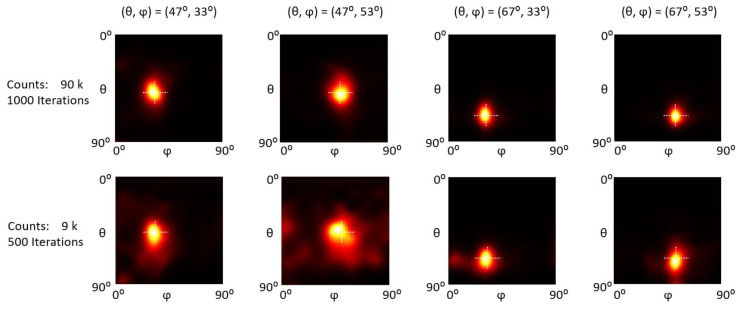
Reconstruction images of a ^60^Co point sources at different positions with different count levels. The intersections of the dotted lines indicate the actual positions.

Figure 9, Figure 10 and Figure 11 show the reconstructed images of representative ^57^Co, ^137^Cs, and ^60^Co single point sources at (47°, 33°), (47°, 53°), (67°, 33°), and (67°, 53°). For each count level, we empirically chose an optimal iteration number (as indicated in the first column in Figure 9, Figure 10 and Figure 11) that yields the best trade-off between image detail and noise. Visual inspections show that all the point sources are accurately located. 

To quantitatively evaluate the positioning accuracy, we calculated the centroid of the point source image as the estimated gamma source position and then defined the positioning bias as the absolute distance between the estimated and true (*θ*, *φ*) positions. We reconstructed ten statistically independent projections with different counts for each testing position and calculated the mean and standard deviation of positioning bias. 

**Figure 12:**
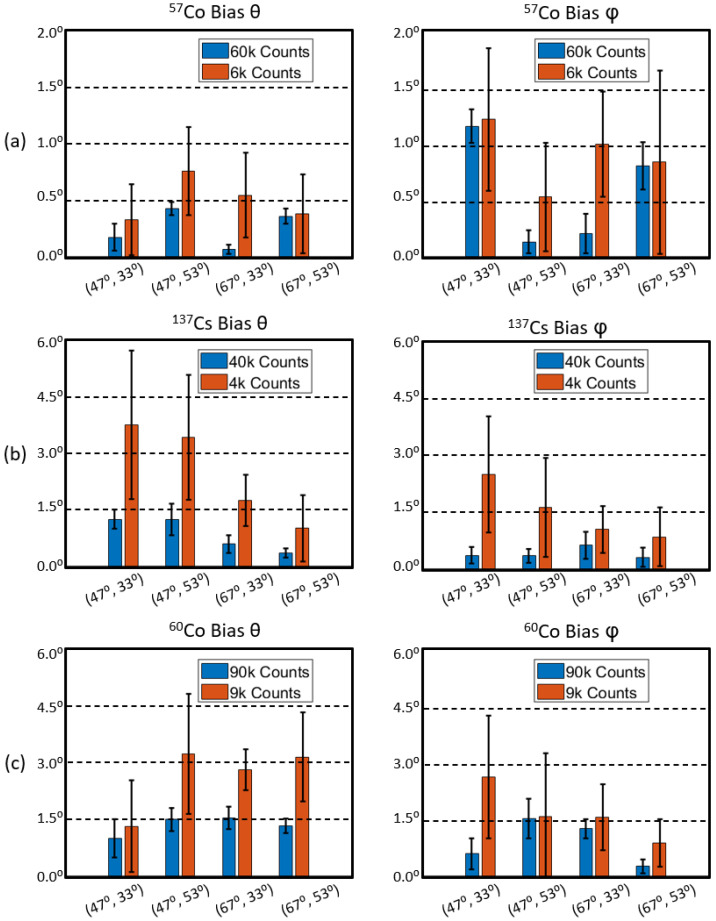
Positioning bias of *θ* and *φ* for locating (**a**) ^57^Co, (**b**) ^137^Cs, and (**c**) ^60^Co point sources with different count levels.

The positioning bias evaluation results are summarized in Figure 12. For ^57^Co sources, the average positioning bias for (*θ*, *φ*) is (0.26° ± 0.16°, 0.58° ± 0.46°) and (0.50° ± 0.38°, 0.90° ± 0.64°) with 60 k and 6 k counts. For ^137^Cs sources, the average positioning bias for (*θ*, *φ*) is (0.86° ± 0.49°, 0.42° ± 0.28°) and (2.49° ± 1.78°, 1.51° ± 1.27°) with 40 k and 4 k counts. For ^60^Co sources, the average positioning bias for (*θ*, *φ*) is (1.35° ± 0.40°, 0.92° ± 0.62°) and (2.64° ± 1.39°, 1.67° ± 1.39°) with 90 k and 9 k counts.

### 4.3. Multiple Point Sources Imaging

**Figure 13:**
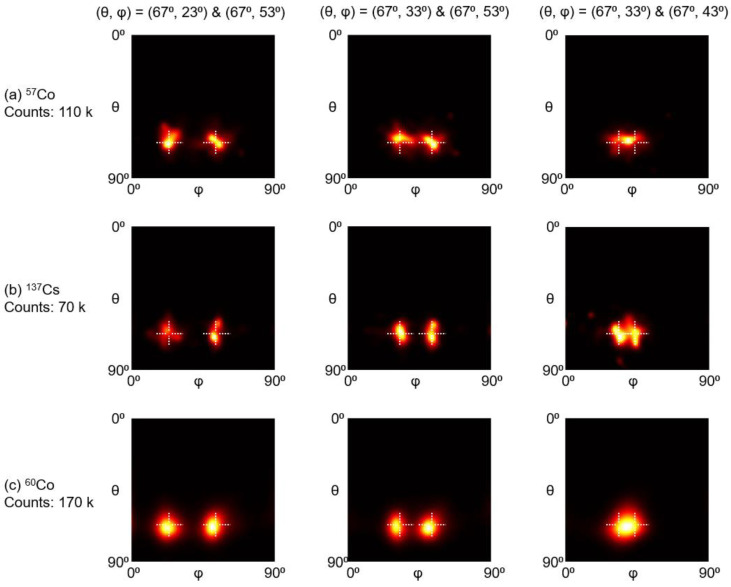
Reconstruction images of two (**a**) ^57^Co, (**b**) ^137^Cs, and (**c**) ^60^Co point sources at different distances.

Figure 13 shows the image reconstruction results of two point sources at different distances. We applied 2000 MLEM iterations for image reconstruction. Two ^137^Cs point sources with 10° separation can be resolved, and two ^57^Co and ^60^Co point sources with 20° separation can be separated, which indicates the proposed gamma camera achieves good angular resolution.

**Figure 14:**
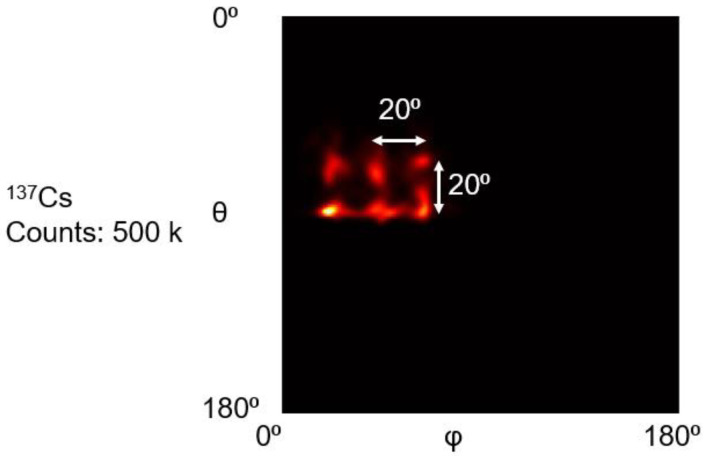
The reconstructed images of experimental 2 × 3 ^137^Cs point source array.

Figure 14 shows the reconstruction results of a 2 × 3 array of ^137^Cs point sources. With 2000 MLEM iterations, all the sources are recognizable in the reconstruction images.

## 5. Discussion

We propose a novel 4π-view gamma camera design with a wide imageable energy range in this work. The gamma camera is composed of interspaced bar-shaped position-sensitive scintillators and heavy metal. With this design, the absorbed gamma events distribution in the gamma camera is sensitive to the incoming gamma rays’ direction. The embedded heavy metal elements enhance collimation for high-energy gamma photons. We implemented this concept using GAGG (Ce) crystals and tungsten bars in a gamma camera prototype. Experimental results show that the proposed gamma camera achieves < 3° positioning accuracy and 10°~20° dual-point-source separation ability with ^57^Co, ^137^Cs, and ^60^Co point sources.

Our group investigated several potential methods of industrial gamma imaging with position-sensitive scintillators. Previously proposed gamma cameras are composed of scintillator-only designs, including monolithic scintillators [11], uniformly distributed scintillators [27,28], or mosaic-patterned scintillators [12]. These designs achieve excellent imaging performance for ^99m^Tc (140 keV) sources and promising results for ^18^F (511 keV) and ^137^Cs (662 keV) sources. However, this method critically needs the scintillator’s collimation for gamma photons. For a typical portable gamma camera with a compact detector size, previously proposed methods are less effective for imaging isotopes with higher gamma energy, such as ^60^Co (1173 and 1333 keV), due to the scintillator’s lower stopping power. The new design in this study, i.e., embedding heavy metal bars into interspaced crystals, successfully enhances the high-energy imaging performance. Compared with existing industrial gamma cameras, such as heavy metal coded-aperture gamma cameras and Compton cameras, the merit of the proposed design achieves advantageous comprehensive performance, including 4π imaging FOV, compact design, high resolution, and wide energy range. The reported resolution in terms of dual-point-separation is 10° for ^137^Cs, compared with 20°–25° separation in [6,29]. For ^60^Co imaging, the proposed device can separate two point sources with a 20° distance. In comparison, most existing studies report single-point-source imaging ability.

The energy resolution of the proposed device needs to be further improved. Potential works to refine the energy resolution include: (1) Regarding the dual-end-readout signal readout strategy and the gain difference between the two SiPMs, in the future we plan to perform fine calibrations of the SiPM gains to correct this. (2) The mismatched emission spectrum of GAGG (Ce) crystal (peaked at 520 nm) and the photodetection efficiency of SiPM (peaked at 420 nm) can be ameliorated by using other SiPM models, such as KETEK PA3325-WB and Hamamatsu S13361-3050, with the photodetection efficiency peaked at 450 nm [30].

One important application of the gamma camera is to simultaneously image multiple sources of different energy. In principle, our system is applicable in this scenario since the detector works in photon-counting mode, and signal processing of each individual event is performed independently. Therefore, it is feasible to create multiple energy-windows channels, and generate projections and performance image reconstructions for each source independently, as long as the total count rate is within the operable range for the detector. In our future work, we will explore the efficacy of simultaneously imaging multiple sources with different gamma ray energies.

## 6. Conclusions

In this work, we developed a 4π-view gamma camera prototype using interspaced bar-shaped GAGG (Ce) crystals and embedded tungsten bars aiming at achieving satisfactory imaging performance in a wide gamma energy range. With imaging experiments of ^57^Co, ^137^Cs, and ^60^Co point sources, we show that the developed gamma camera achieves a positioning accuracy of <3° for all gamma energies. It can clearly resolve two ^137^Cs point sources with 10° separation, two ^57^Co and two ^60^Co point sources with 20° separation, as well as a 2 × 3 ^137^Cs point source array with 20° separation. We conclude that the proposed gamma camera design significantly improves the comprehensive imaging performance in a wide energy range without sacrificing FOV size and system compactness. It has promising potential in nuclear security applications.

## Figures and Tables

**Table 1:** Experimental setup.

Point Source Type	^57^Co	^137^Cs	^60^Co
Point Source Activity	5.2 mCi	11.2 mCi	4.6 mCi
Source-Detector Distance	1 m	1 m	1 m
Energy Window	96–146 keV	530–794 keV	938–1600 keV
Measurement interval	5°	5°	10°
Measurement points	19 (θ) × 37 (φ)	19 (θ) × 37 (φ)	10 (θ) × 19 (φ)
Time at Each Position	15 s	20 s	150 s
Counts at Each Position	100 k	100 k	600 k
Counting Rates	34.7 cps/MBq	12.1 cps/MBq	23.5 cps/MBq
